# Editorial: New Perspectives to Unlock the Current Impasse in Treating Anorexia Nervosa

**DOI:** 10.3389/fpsyg.2020.00207

**Published:** 2020-02-18

**Authors:** Emilio Gutierrez, C. Laird Birmingham

**Affiliations:** ^1^Departamento de Psicología Clínica y Psicobiología and Unidad Venres Clínicos, Facultad de Psicología, Universidad de Santiago, Santiago de Compostela, Spain; ^2^Department of Psychiatry, University of British Columbia, Vancouver, BC, Canada

**Keywords:** translational research, animal research, hyperactivity, causation, evidence-based treatments

Regardless of current treatments, the mortality rate from anorexia nervosa has remained constant (Birmingham et al., 2005; Signorini et al., 2007). Besides family therapy for medically stable adolescents, either in conjoint or separated formats (Fisher et al., 2018), the current spectrum of evidence-based AN treatments ranges from specialized brand type treatments (enhanced-cognitive behavior, psychodynamic, and interpersonal therapy) to new faceted treatments customized to tap the specific characteristics of patients, as is the case of the Maudsley Model of Anorexia Treatment in Adults, MANTRA (Schmidt et al., 2012). However, several randomized control trials have shown the “dodo verdict” prevails since none of these treatments was superior to the others (Gutiérrez and Carrera, 2018; van den Berg et al., 2019). Furthermore, this “tie score” remains constant when the aforementioned treatments are compared either with the Specialized Clinical Support Management, which was originally conceived as a treatment control condition not based on any “theoretical model of causation or strategies based on theory” (Jordan et al., 2019), or with treatment as usual (Zipfel et al., 2014). In this special edition, Brodrick et al. reports that the current treatment for patients in Texas with severe eating disorders is neither effective nor individualized.

Pharmacological treatment has failed to offer a more optimistic picture regarding AN treatment (Davis and Attia, 2017). However, at variance with pharmacotherapy whose action is more loosely connected to aetiological or maintenance factors, psychological treatments mainly focus on different conceptualizations of AN. Nevertheless, provided that the aims of treatment are a logical corollary of the basic understanding of a disorder (Mayer, 1963), current “tie score” among psychological treatments for AN does not support the underlying principles of one treatment over other treatments. Therefore, without excluding other possibilities, it is not preposterous to think that either the treatments developed in the last four decades have followed a misleading path or they have undermined the relevance of other signs and symptoms of AN (Gutiérrez and Carrera, 2014).

Nowadays, we too readily believe that treatments from previous eras (Lucas, 1986) were misguided and ineffective, or even iatrogenic owing to their conceptualizations of AN (i.e., oral impregnation fantasies). Our colleagues from Sweden (Södersten et al.) offer a radical departure from mainstream thinking by suggesting that the assumption that body-image disturbance is a key causal agent of AN has skewed our understanding of hyperactivity as a core feature for both diagnosis and treatment. In this regard, Casper in line with previous enlightening contributions (Casper, 1998, 2006) invites us to consider restlessness and continued motor activity as constitutive of AN phenotype.

Hyperactivity has been repeatedly reported in AN by clinicians and researchers since the first modern descriptions of the illness. In spite of excessive activity being considered a fundamental clinical feature (Kron et al., 1978) even before the DSM-III was released (American Psychiatric Association, 1980), hyperactivity has been traditionally assigned a secondary role in AN diagnosis. Similar to Cinderella not being invited to the ball in the popular fairytale, hyperactivity has been unjustly neglected as a relevant criterion for the diagnosis of AN in the DSM series (Gutierrez et al., 2002). This disregard for hyperactivity is bewildering since it is a stable sign universally recognized for more than 70 years before the current conception of AN with its emphasis on distorted body image, and weight phobia. Furthermore, the neglect of frantic hyperactivity in AN contradicts research showing that excessive activity often precedes the onset of the disorder (Davis et al., 2005), as first pointed out by Janet who stated that “the exaggeration of the movement is sometimes anterior to the refusal of food and therefore precedes all these reasonings” (Janet, 1901, p. 501). Furthermore, Charles Lasègue, in the first case of AN described in detail in the literature, underscored that the abstinence from food “tends to increase the aptitude for movement” (Lasègue, 1873, p. 266). Moreover, early in the literature Janet acknowledged the negative impact of excessive activity upon eating by stating “The exaltation of the strength, the feeling of euphoria, as it is known in the ecstatic saints, for instance, does away with the need of eating” (Janet, 1907, p. 242).

Hyperactivity is also a central topic in another paper (Hebebrand et al.), namely the use of off-label treatment with metreleptin to neutralize the reward inherent in the vicious cycle of restrictive eating and excessive activity in AN patients.

Phenotypic and genotypic subtypes of AN have been reported by Sala et al., who studied a single nucleotide polymorphism in the oxytocin receptor gene; and Rohde et al. reported an operationalized psychodynamic personality diagnosis in eating disorders in order to capture the personality profile of restrictive and purging subtypes of AN in order to preserve an AN entity distinct from bulimia nervosa (Birmingham et al., 2009).

The evidence coming from Sydney (Kezelman et al.) regarding the hormonal and psychological consequences of rapid refeeding is also compelling. Recent attention to post-prandial anxiety has revealed a significant reduction in patients resting for half an hour immediately after lunch in a room at 32°C (Zandian et al., 2017), the same ambient temperature which reverses activity-based anorexia (Gutierrez, 2013). It is worth noting that, “to supply external heat as well as food to patients” was the very first recommendation by Gull (1874), based on the observations of the effects of heat on starved animals. Moreover, grounded in an evolutionary perspective, Scolnick offers clues to understand the pathogenic process in AN, departing from the perspective of extreme metabolic adaptation to food depletion and cold in hibernating mammals.

As with Cinderella, the ball was far from over for hyperactivity, which should gain acceptance, not only in terms of its relevance for the diagnosis of AN diagnosis, but also in treatment.

## Author Contributions

All authors listed have made a substantial, direct and intellectual contribution to the work, and approved it for publication.

### Conflict of Interest

The authors declare that the research was conducted in the absence of any commercial or financial relationships that could be construed as a potential conflict of interest.

## References

[B1] American Psychiatric Association (1980). Diagnostic and Statistical Manual of Mental Disorders, 3rd Edn. Washington, DC: American Psychiatric Publishing.

[B2] Birmingham C. L. Su J. Hlynsky J. A. Goldner E. M. Gao M. (2005). The mortality rate from anorexia nervosa. Int. J. Eat. Disord. 38, 143–146. 10.1002/eat.20164 16134111

[B3] Birmingham C. L. Touyz S. Harbottle J. (2009). Are anorexia nervosa and bulimia nervosa separate disorders? Challenging the ‘transdiagnostic’ theory of eating disorders. Eur. Eat. Disord. Rev. 17, 2–13. 10.1002/erv.896 18781580

[B4] Casper R. C. (1998). Behavioral activation and lack of concern, core symptoms of anorexia nervosa? Int.J.Eat. Disord. 24, 381–393. 9813763 10.1002/(sici)1098-108x(199812)24:4<381::aid-eat5>3.0.co;2-q

[B5] Casper R. C. (2006). The ‘drive for activity’ and “restlessness” in anorexia nervosa: potential pathways. J. Affect. Disord. 92, 99–107. 10.1016/j.jad.2005.12.039 16448703

[B6] Davis C. Blackmore E. Katzman D. K. Fox J. (2005). Female adolescents with anorexia nervosa and their parents: a case-control study of exercise attitudes and behaviours. Psychol. Med. 35, 377–386. 10.1017/S0033291704003447 15841873

[B7] Davis H. Attia E. (2017). Pharmacotherapy of eating disorders. Curr. Opin. Psychiatry 30, 452–457. 10.1097/YCO.0000000000000358 28806268 PMC8221404

[B8] Fisher C. A. Skocic S. Rutherford K. A. Hetrick S. E. (2018). Family therapy approaches for anorexia nervosa. Cochrane Database Syst. Rev. 10:CD004780. 10.1002/14651858.CD004780.pub3. 30320438 PMC6517149

[B9] Gull W. (1874). Anorexia nervosa (apepsia hysterica, anorexia hysterica). Trans. Clin. Soc. Lond. 7, 22–28.

[B10] Gutierrez E. (2013). A rat in the labyrinth of anorexia nervosa: contributions of the activity-based anorexia rodent model to the understanding of anorexia nervosa. Int.J.Eat. Disord. 46, 289–301. 10.1002/eat.22095 23354987

[B11] Gutiérrez E. Carrera O. (2014). Psychotherapy in anorexia nervosa: what does the absence of evidence mean? World J. Transl. Med. 3, 150–157. 10.5528/wjtm.v3.i3.150

[B12] Gutiérrez E. Carrera O. (2018). Anorexia nervosa treatments and Occam's razor. Psychol. Med. 48, 1390–1391. 10.1017/S0033291717003944 29366435

[B13] Gutierrez E. Vazquez R. Beumont P. J. V. (2002). Do people with anorexia nervosa use sauna baths? A reconsideration of heat treatment in anorexia nervosa. Eat. Behav. 3, 133–142. 10.1016/S1471-0153(01)00051-4 15001010

[B14] Janet P. (1901). La maladie du scrupule ou l'aboulie délirante: Le contenu des obsessions. Rev. Philos. Fr.'Étrang. 51, 499–524.

[B15] Janet P. (1907). The Major Symptoms of Hysteria. New York, NY: Macmillan.

[B16] Jordan J. McIntosh V. V. Bulik C. M. (2019). Specialist Supportive Clinical Management for anorexia nervosa: what it is (and what it is not). Austral. Psychiatry. [Epub ahead of print]. 10.1177/1039856219875024 31523976

[B17] Kron L. Katz J. L. Gorzynski G. Weiner H. (1978). Hyperactivity in anorexia nervosa: a fundamental clinical feature. Comp. Psychiat. 19, 433–440. 679677 10.1016/0010-440x(78)90072-x

[B18] Lasègue C. (1873). De l'anorexie hystérique. Archiv. Gen. Méd. 21, 385–403.

[B19] Lucas A. R. (1986). Anorexia nervosa; historical background and biopsychosocial determinants. Semin. Adolesc. Med. 16, 1–9. 3299571

[B20] Mayer J. (1963). Anorexia nervosa. Postgrad. Med. 34, 529–534. 10.1080/00325481.1963.11694920 14054626

[B21] Schmidt U. Oldershaw A. Jichi F. Sternheim L. Startup H. McIntosh V.. (2012). Out-patient psychological therapies for adults with anorexia nervosa: randomised controlled trial. Br. J. Psychiatry 201, 392–399. 10.1192/bjp.bp.112.112078 22995632

[B22] Signorini A. de Filippo E. Panico S. de Caprio C. Pasanisi F. Contaldo F. (2007). Long-term mortality in anorexia nervosa: a report after an 8-year follow-up and a review of the most recent literature. Eur. J. Clin. Nutr. 61, 119–122. 10.1038/sj.ejcn.1602491 16885933

[B23] van den Berg E. Houtzager L. de Vos J. Daemen I. Katsaragaki G. Karyotaki E.. (2019). Meta-analysis on the efficacy of psychological treatments for anorexia nervosa. Eur. Eat. Disord. Rev. 27, 331–351. 10.1002/erv.2683 31124215

[B24] Zandian M. Holmstedt E. Larsson A. Bergh C. Brodin U. Södersten P. (2017). Anxiolytic effect of warmth in anorexia nervosa. Acta Psychiatr. Scand. 135, 266–267. 10.1111/acps.12691 28043086

[B25] Zipfel S. Wild B. Gro ß, G. Friederich H. C. Teufel M. Schellberg D.. (2014). Focal psychodynamictherapy, cognitive behaviour therapy, and optimised treatment as usual in outpatients with anorexia nervosa (ANTOP study): randomised controlled trial. Lancet 383, 127–137. 10.1016/S0140-6736(13)61746-8 24131861

